# A study on the safety and efficacy of reveglucosidase alfa in patients with late-onset Pompe disease

**DOI:** 10.1186/s13023-017-0693-2

**Published:** 2017-08-24

**Authors:** Barry J. Byrne, Tarekegn Geberhiwot, Bruce A. Barshop, Richard Barohn, Derralynn Hughes, Drago Bratkovic, Claude Desnuelle, Pascal Laforet, Eugen Mengel, Mark Roberts, Peter Haroldsen, Kristin Reilley, Kala Jayaram, Ke Yang, Liron Walsh, on behalf of the POM-001/002 Investigators

**Affiliations:** 1 University of Florida, School of Medicine, 1600 SW Archer Road, Gainesville, FL 32607 USA; 2 University Hospital Birmingham, Mindelsohn Way, Edgbaston, Birmingham, B15 2GW UK; 3 University of California San Diego Health System, 4168 Front Street, San Diego, CA 92103 USA; 4 Kansas University Medical Center, 3901 Rainbow Blvd/MSN 2012, Kansas City, KS 66160 USA; 5 Royal Free London NHS Foundation & University College London Department of Hematology, Pond St, London, NW3 2QG UK; 6 SA Pathology, Frome Rd, Adelaide, SA 5000 Australia; 7 University Hospital of Nice, Pasteur Hospital, Nice, France; 8 Paris-Est Neuromuscular Center, INSERM U974, UPMC, Hôpital Pitié-Salpêtrière, 47-83 boulevard de l’Hôpital, 75013 Paris, France; 9 Johannes Gutenberg University, Langenbeckstr. 1, 55131 Mainz, Germany; 10 Salford Royal NHS Foundation Trust, M6 8HD, Salford, UK; 11 BioMarin Pharmaceutical, 105 Digital Drive, Novato, CA 94949 USA; 12 Department of Pediatrics, University of Florida, P.O. Box 100296, Gainesville, FL 32610 USA

**Keywords:** Reveglucosidase alfa, Late-onset Pompe disease, Enzyme replacement therapy, Pharmacokinetics, Safety, Efficacy, Respiratory, Pulmonary

## Abstract

**Background:**

Late-onset Pompe disease is a rare genetic neuromuscular disorder caused by lysosomal acid alpha-glucosidase (GAA) deficiency that ultimately results in mobility loss and respiratory failure. Current enzyme replacement therapy with recombinant human (rh)GAA has demonstrated efficacy in subjects with late-onset Pompe disease. However, long-term effects of rhGAA on pulmonary function have not been observed, likely related to inefficient delivery of rhGAA to skeletal muscle lysosomes and associated deficits in the central nervous system. To address this limitation, reveglucosidase alfa, a novel insulin-like growth factor 2 (IGF2)-tagged GAA analogue with improved lysosomal uptake, was developed. This study evaluated the pharmacokinetics, safety, and exploratory efficacy of reveglucosidase alfa in 22 subjects with late-onset Pompe disease who were previously untreated with rhGAA.

**Results:**

Reveglucosidase alfa plasma concentrations increased linearly with dose, and the elimination half-life was <1.2 h. Eighteen of 22 subjects completed 72 weeks of treatment. The most common adverse events were hypoglycemia (63%), dizziness, fall, headache, and nausea (55% for each). Serious adverse events included hypersensitivity (*n* = 1), symptomatic hypoglycemia (*n* = 2), presyncope (*n* = 1), and acute cardiac failure (*n* = 1). In the dose-escalation study, all treated subjects tested positive for anti-reveglucosidase alfa, anti-rhGAA, anti-IGF1, and anti-IGF2 antibodies at least once. Subjects receiving 20 mg/kg of reveglucosidase alfa demonstrated increases in predicted maximum inspiratory pressure (13.9%), predicted maximum expiratory pressure (8.0%), forced vital capacity (−0.4%), maximum voluntary ventilation (7.4 L/min), and mean absolute walking distance (22.3 m on the 6-min walk test) at 72 weeks.

**Conclusions:**

Additional studies are needed to further assess the safety and efficacy of this approach. Improvements in respiratory muscle strength, lung function, and walking endurance in subjects with LOPD may make up for the risk of hypersensitivity reactions and hypoglycemia. Reveglucosidase alfa may provide a new treatment option for patients with late-onset Pompe disease.

**Trial registration:**

ISRCTN01435772 and ISRCTN01230801, registered 27 October 2011.

**Electronic supplementary material:**

The online version of this article (doi:10.1186/s13023-017-0693-2) contains supplementary material, which is available to authorized users.

## Background

Late-onset Pompe disease (LOPD), a rare genetic neuromuscular disorder, results in severe respiratory dysfunction that often progresses to respiratory failure [1]. LOPD is caused by a deficiency of the lysosomal enzyme acid alpha glucosidase (GAA) [2], which prevents enzymatic breakdown of glycogen. As a result, glycogen accumulates in multiple tissues. The most severe pathology in LOPD patients is observed in skeletal muscles [3, 4]. Myopathy in proximal skeletal muscles, for example, reduces mobility in patients and eventually leads to loss of independent ambulation [5]. Furthermore, weakness of the respiratory muscles (eg, diaphragm) causes respiratory dysfunction that initially manifests as sleep-disordered breathing and nighttime hypercapnia [6]. In more than 70% of patients, respiratory dysfunction progresses to respiratory failure, which is the most common cause of mortality in LOPD [7]. The early onset and progressive nature of the disease highlight the need for safe and highly effective treatments.

Recombinant human GAA (rhGAA), the first enzyme replacement therapy (ERT) for LOPD, became available in 2006 [8–11]. Although treatment with rhGAA reduces mortality in infants with Pompe disease, it provides limited improvements in mobility and respiratory function [9, 12–14]. The uptake of endogenous GAA into tissues is mediated by the binding of bis-mannose 6-phosphorylated moieties on GAA to the cation-independent mannose 6-phosphate receptors (CI-MPR) in tissues [15]. However, rhGAA binds to the CI-MPR with low affinity [16–18], which reduces uptake of the recombinant enzyme into lysosomes.

To enhance the uptake of GAA into the lysosome, a CI-MPR-targeting peptide—derived from insulin-like growth factor 2 (IGF2)—was fused to GAA to form the novel chimeric enzyme reveglucosidase alfa (glycosylation-independent lysosomal targeting [GILT]-tagged rhGAA). In preclinical models of Pompe disease, reveglucosidase alfa is taken up by skeletal muscle cells with greater efficiency than rhGAA [15] and is more effective at reducing glycogen in skeletal muscle [19]. The improved efficacy in nonclinical models prompted an initial evaluation of reveglucosidase alfa in LOPD subjects [19]. Here we report the results of a first-in-human, phase 1/2, open-label clinical trial that evaluated the pharmacokinetics (PK), safety, tolerability, and efficacy of reveglucosidase alfa in ambulatory LOPD subjects who were naïve to prior ERT (ie, previously untreated) and had mild-to-moderate respiratory impairment.

## Methods

### Study design

This ongoing phase 1/2, international, multicenter, open-label clinical trial of reveglucosidase alfa initially enrolled subjects in a dose-escalation study (Clinicaltrials.gov identifier: NCT01230801; POM-001) [20]. Subjects could then continue treatment in a dose-extension study (Clinicaltrials.gov identifier: NCT01435772; POM-002) [21]. POM-001 consisted of a 25-day pretreatment screening period, a 3-day baseline and enrollment period, a 24-week treatment period, and a provision to continue participation in the dose-extension study. Following the screening and baseline assessments, subjects were treated every 2 weeks for the 24-week treatment period. After completion of POM-001, eligible subjects continued reveglucosidase alfa treatment in POM-002 for additional 24-week treatment cycles (up to a maximum of twenty 24-week cycles, or 480 weeks). Local review boards, ethics committees, and health authorities at each of the 12 study centers approved the protocol and all amendments. Informed consent was obtained from each subject’s parent/guardian, and assent was obtained from each subject where appropriate.

### Subject selection

All eligible subjects had a confirmed diagnosis of LOPD based on 2 GAA gene mutations and endogenous GAA activity <75% of the lower limit of the normal adult range. Additional inclusion criteria included age ≥ 13 years at the time of study enrollment, predicted upright forced vital capacity (FVC) ≥30%, and either predicted upright FVC <80% or a reduction in supine FVC >10% compared with upright FVC. Eligible subjects were naïve to ERT with rhGAA (ie, previously untreated) and must have been able to ambulate at least 40 m on the 6-min walk test (6MWT). Subjects were excluded if they had previously received any experimental or approved therapy for LOPD prior to enrollment and had a medical condition that, in the opinion of investigators, might compromise the subject’s ability to comply with the study protocol requirements.

### Treatment

During the dose-escalation study (POM-001), subjects were sequentially enrolled in 1 of 3 dosing regimens of reveglucosidase alfa (5 mg/kg [*n* = 3], 10 mg/kg [*n* = 3], and 20 mg/kg [*n* = 20] administered as an intravenous infusion (of approximately 1.5–4 h’s duration) every 2 weeks during the 24-week treatment cycle. Interim safety assessments were completed by an independent review board prior to any dose escalation. POM-002, the extension phase, enrolled subjects from POM-001 who continued treatment at 5 mg/kg, 10 mg/kg, or 20 mg/kg levels for additional treatment cycles. Treatment of patients was completed, and the analysis included data for three 24-week treatment cycles (ie, 72 weeks of total exposure to reveglucosidase alfa).

### Pharmacokinetic analyses

Reveglucosidase alfa PK was evaluated (at day 1, week 12, and week 24) in all 3 dosing cohorts, using blood samples taken before, during, and after infusions at predose, 1 h into infusion, 2 h into infusion, end of infusion (EOI; time 0 for PK), and 0.25, 0.5, 1.0, 2.0, 3.0, 6.0, 12, 18, and 24 h post EOI.

### Safety assessment

Infusion-related reactions were defined as adverse events (AEs) occurring within 1 day after infusion. Assessments included physical examinations and vital sign assessments, pulse oximetry and blood glucose monitoring before, during, and after infusions, AE recordings (including signs of hypersensitivity and hypoglycemia), chest X-rays, 12-lead echocardiograms, clinical laboratory assessments, and antibody testing (anti-reveglucosidase alfa, anti-GAA, anti-insulin-like growth factor 1 [IGF1], and anti-IGF2). Serum IGF analytes (IGF1, IGF2, and insulin-like growth factor-binding protein-3) were measured to assess the effect of reveglucosidase alfa on serum IGF levels. A subject who was clinically symptomatic or whose blood glucose level was <60 mg/dL was treated as appropriate (oral and intravenous [IV] dextrose). Subjects were measured every 15 min after treatment for hypoglycemia until their blood glucose reached >100 mg/dL.

### Efficacy evaluation

The efficacy of reveglucosidase alfa was evaluated at baseline and following 24 and 72 weeks of treatment. Evaluations included mobility/endurance (6MWT), respiratory muscle strength (maximum inspiratory pressure [MIP], maximum expiratory pressure [MEP]), and lung function tests (FVC-upright, FVC-supine, maximum voluntary ventilation [MVV]). Efficacy from the 20 mg/kg group is presented in this manuscript.

### Data analysis

The extension phase (POM-002) has recently been completed. The data presented are from the 72-week interim analysis. The efficacy analysis population consisted of all subjects who were dosed with 20 mg/kg reveglucosidase alfa. The safety analysis population consisted of all subjects who enrolled into POM-001 and were dosed. Safety analyses included a summary of AEs and immunogenicity. The statistical significance of treatment effects was not determined as no pre-planned statistical analyses were conducted for this study. Graphical displays were provided for efficacy endpoints.

## Results

### Study subjects

**Fig. 1:**
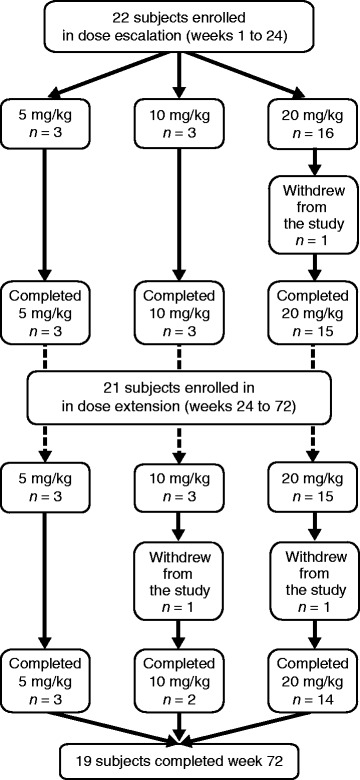
Subject flow. Patient withdrawals during dose-escalation (POM-001) and dose-extension (POM-002) were by physician decision and subject decision, respectively

All enrolled subjects (*N* = 22) had a confirmed diagnosis of LOPD based on 2 GAA gene mutations (see Additional file 1) and were previously untreated with rhGAA. The mean (range) % lower limit of normal GAA activity was 20.37 (5.5, 58.3). Subjects included in the intent-to-treat population (Fig. 1) were administered reveglucosidase alfa at initial doses of 5, 10, and 20 mg/kg, (*n* = 3, *n* = 3, *n* = 16, respectively), every 2 weeks for 24 weeks. For the dose-escalation phase (POM-001), 21 subjects completed 24 weeks of dosing and enrolled in the extension study (POM-002). In the first 72 weeks, 3 subjects withdrew from the study because of a hypersensitivity AE (2 subjects in the 20 mg/kg cohort and 1 in the 10 mg/kg cohort). One subject in the 5 mg/kg cohort discontinued treatment because of a hypersensitivity AE but remained in the study. Eighteen subjects received reveglucosidase alfa (5, 10, or 20 mg/kg) every 2 weeks for up to a total of 72 weeks. Demographics and baseline characteristics (Table 1) describe a cohort of ambulatory LOPD subjects who had moderate respiratory impairment (mean ± standard deviation [SD] percent predicted FVC, 60.9 ± 19.2; MIP, 40.3 ± 24.0; and MEP, 35.5 ± 14.4), with limitations for endurance seen at baseline.

**Table 1:** Demographics and baseline characteristics

	POM-001 (*N* = 22)
Mean age (range), years	49.3 (28–58)
Gender, *n* (%)	
Male	14 (64)
Female	8 (36)
Mean weight (range), kg	89.0 (49.2–144.5)
Mean time since initial diagnosis (range), years	5.4 (0.106–25.12)
Mean % predicted MIP ± SD	40.3 ± 24.0
Mean % predicted MEP ± SD	35.5 ± 14.4
Mean MVV ± SD, L/min	68.9 ± 27.5
Mean % predicted FVC upright ± SD	60.9 ± 19.2
6MWT ± SD, meters	352.5 ± 151.1

*Abbreviations:*

*6MWT* 6-min walk test, *FVC* forced vital capacity, *MEP* maximal expiratory pressure, *MIP* maximal inspiratory pressure, *MVV* maximum voluntary ventilation, *SD* standard deviation

### Pharmacokinetics

**Fig. 2:**
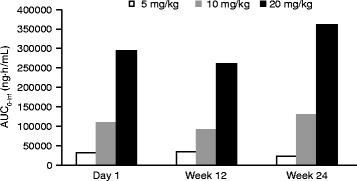
Reveglucosidase alfa exposure-dose linearity over 24 weeks of treatment. Exposure data presented as AUC_0-inf_ on day 1, week 12, and week 24 of dosing. Abbreviation: AUC_0-inf_, area under the concentration-time curve from 0 h extrapolated to infinity

Reveglucosidase alfa was rapidly eliminated from plasma with a mean terminal half-life (t_½_) of <1.2 h post infusion for the 3 cohorts on day 1, week 12, and week 24 (Table 2). Following 24 weeks of reveglucosidase alfa treatment, mean exposure by area under the concentration-time curve (AUC) increased by 18 and 23% in the 10 and 20 mg/kg arms respectively, and mean exposure by AUC decreased by 30% in the 5 mg/kg arm. Mean clearance (CL) values ranged from 73.5–184 mL/h·kg in 10 and 20 mg/kg subjects over the 24-week course of treatment and demonstrated slight decreases (10 mg/kg, −22.4%; 20 mg/kg, −13.5%) from day 1 to week 24. Over the 24-week course of treatment in 5 mg/kg subjects, mean t_½_ increased ≈2.0-fold, CL increased by 3.0-fold, and volume in the terminal state and in steady state increased 7.5- and 5.0-fold, respectively. Increases in the mean values of AUC from 0 h and extrapolated to infinity (AUC_0-inf_) were greater than dose proportional (>1:1) and dose linear (R^2^ > 0.99) on the 3 PK evaluation days (day 1, week 12, and week 24), which indicate exposure to reveglucosidase alfa is linear over the 4.0-fold dose range (5–20 mg/kg) and over the 24 weeks of treatment (Fig. 2).

**Table 2:** Reveglucosidase alfa mean (range) PK parameters in LOPD subjects^a^

	5 mg/kg			10 mg/kg			20 mg/kg		
	Day 1	Week 12	Week 24	Day 1	Week 12	Week 24	Day 1	Week 12	Week 24
Mean PK parameter	(*n* = 3)	(*n* = 3)	(*n* = 3)	(*n* = 3)	(*n* = 3)	(*n* = 3)	(*n* = 10)	(*n* = 9)	(*n* = 9)
C_max_ (range), ng/mL	28,172 (16072–38,300)	28,829 (20072–38,300)	14,117 (3084–30,901)	80,873 (41225–124,800)	49,843 (3235–76,825)	55,187 (3451–100,440)	112,974 (59912–250,500)	87,182 (25197–171,780)	126,196 (53246–218,400)
AUC_0-inf_ (range), ng·h/mL	32,004 (19865–43,524)	34,411 (21470–44,478)	22,343 (4587–52,228)	109,655 (58114–143,692)	90,439 (25777–135,436)	129561^b^(95030–164,092)	294,648 (157200–684,264)	261,255 (90854–417,087)	361,068 (109285–597,203)
Vz (range), mL/kg	66.3 (46.0–105)	58.7 (38.5–90.6)	499 (42.3–1083)	98.4 (55.9–121)	442 (42.8–1154)	44.2^b^(31.8–57.0)	76.6 (20.8–177)	95.5 (29.8–180)	92.2 (12.8–273)
CL (range), mL/h·kg	173 (115–252)	160 (112–233)	558 (95.7–1090)	107 (69.6–172)	184 (73.8–388)	83.1^b^(60.9–105)	84.9 (29.2–127)	101 (48.0–220)	73.5 (33.5–183)
Half-life (range), hours	0.261 (0.217–0.289)	0.250 (0.237–0.270)	0.507 (0.306–0.688)	0.701 (0.475–1.07)	1.20 (0.327–2.06)	0.367^b^(0.361–0.373)	0.723 (0.310–2.22)	0.715 (0.343–1.47)	0.767 (0.259–1.65)

*Abbreviations:*

*AUC*_*0-inf*_ area under the concentration-time curve from 0 h extrapolated to infinity, *CL* clearance, *C*_*max*_ minimum concentration, *LOPD* late-onset Pompe disease, *PK* pharmacokinetics, *Vz* volume

^a^LOPD subject cohorts received 5 mg/kg (*n* = 3), 10 mg/kg (*n* = 3), or 20 mg/kg (*n* = 10 at week 1; *n* = 9 at week 24) of reveglucosidase alfa as a weekly 2-h infusion for 24 weeks

^*b*^ *n* = 2 for this parameter

### Safety and tolerability

In general, reveglucosidase alfa was reasonably tolerated in all 22 subjects treated with 5 mg/kg, 10 mg/kg, or 20 mg/kg every other week. AEs that occurred in ≥10% of the subjects are shown in Table 3. The most common AEs were hypoglycemia (63%) and dizziness, fall, headache, and nausea (55% for each). The majority of events were mild or moderate in severity. Three subjects reported 6 events assessed as severe (grade 3) in the context of a hypersensitivity event and included urticaria, rash, pruritis, chest tightness, and allergic reaction. Three subjects reported 4 events of symptomatic hypoglycemia including 3 events reported as severe and 1 reported as life-threatening (grade 4). Other grade 3 events included aggression, presyncope, and venous thrombosis, reported in 3 subjects. Six of the 22 subjects (27%) experienced 8 AEs assessed as serious by the investigator. Of these, 5 were assessed as related to the study drug and included hypoglycemia (*n* = 2, 9%) and acute cardiac failure, hypersensitivity, and presyncope (*n* = 1, 4.5% for each). The subject with acute cardiac failure was hospitalized overnight for a protocol-mandated polysomnography. During the hospital stay, a chest X-ray revealed pulmonary congestion. Concurrent medical conditions included respiratory failure and hypertension. The subject received furosemide, and the event resolved the next day.

**Table 3:** Adverse events occurring in ≥10% LOPD subjects

Subjects with any AE, *n* (%)^a^	5 mg/kg (*N* = 3)	10 mg/kg (*N* = 3)	20 mg/kg (*N* = 16)	Total (*N* = 22)
Hypoglycemia	0 (0)	1 (33)	14 (88)	15 (68)
Dizziness	1 (33)	1 (33)	10 (63)	12 (55)
Fall	3 (100)	3 (100)	6 (38)	12 (55)
Headache	2 (67)	2 (67)	8 (50)	12 (55)
Nausea	1 (33)	1 (33)	10 (63)	12 (55)
Fatigue	2 (67)	1 (33)	7 (44)	10 (45)
Chest discomfort	2 (67)	1 (33)	6 (38)	9 (41)
Vomiting	1 (33)	1 (33)	6 (38)	8 (36)
Dyspnea	0 (0)	1 (33)	6 (38)	7 (32)
Malaise	1 (33)	0 (0)	6 (38)	7 (32)
Chills	0 (0)	1 (33)	5 (31)	6 (27)
Nervousness	1 (33)	0 (0)	5 (31)	6 (27)
Tachycardia	0 (0)	1 (33)	8 (50)	6 (27)
Abdominal pain upper	1 (33)	0 (0)	4 (25)	5 (23)
Feeling hot	0 (0)	1 (33)	4 (25)	5 (23)
Flushing	1 (33)	0 (0)	4 (25)	5 (23)
Rash	1 (33)	0 (0)	4 (25)	5 (23)
Urticaria	1 (33)	1 (33)	3 (19)	5 (23)
Cold sweat	0 (0)	0 (0)	4 (25)	4 (18)
Erythema	1 (33)	0 (0)	3 (19)	4 (18)
Feeling cold	0 (0)	1 (33)	3 (19)	4 (18)
Hyperhidrosis	0 (0)	0 (0)	4 (25)	4 (18)
Hypertension	0 (0)	0 (0)	4 (25)	4 (18)
Hypotension	0 (0)	0 (0)	4 (25)	4 (18)
Edema peripheral	0 (0)	0 (0)	3 (19)	3 (14)
Hot flush	1 (33)	0 (0)	2 (13)	3 (14)
Infusion-related reaction	2 (67)	0 (0)	1 (6)	3 (14)
Pallor	0 (0)	0 (0)	3 (19)	3 (14)
Pruritus	0 (0)	0 (0)	3 (19)	3 (14)
Rash pruritic	1 (33)	1 (33)	1 (6)	3 (14)
Tremor	0 (0)	0 (0)	3 (19)	3 (14)
Subjects with any serious AE, *n* (%)				
Hypoglycemia	0 (0)	0 (0)	2 (13)	2 (9)
Acute cardiac failure	0 (0)	0 (0)	1 (6)	1 (5)
Hypersensitivity	0 (0)	0 (0)	1 (6)	1 (5)
Presyncope	0 (0)	0 (0)	1 (6)	1 (5)

*Abbreviations:*

*AE* adverse event, *LOPD* late-onset Pompe disease

^a^All 22 subjects reported ≥1 AE

Hypoglycemia, a potential pharmacologic effect of the IGF2 moiety of reveglucosidase alfa, was dose related. AEs of hypoglycemia were reported in 0/3 (0%) subjects receiving 5 mg/kg, 1/3 (33%) receiving 10 mg/kg, and 14/16 (88%) receiving 20 mg/kg. Hypoglycemia AEs generally occurred intermittently during and/or within 2 h after the EOI and were managed with caloric supplementation shortly after occurrence. Based on glucose measures taken pre-infusion and up to 8 h post infusion, the majority of the hypoglycemia AEs were reported as grade 1 and 2, and were nonserious. Of the 15 subjects who experienced a hypoglycemic AE, 2 subjects who received 20 mg/kg reveglucosidase alfa each experienced 1 serious hypoglycemia AE. One subject experienced a grade 3 serious AE of symptomatic hypoglycemia (blood glucose, 36 mg/dL; diaphoresis, shaking, flushed, and light headed), 3 h following initiation of the week 35 infusion of reveglucosidase alfa. Another subject experienced a grade 4 serious AE of symptomatic hypoglycemia (blood glucose, 29 mg/dL; clammy and sweaty, with blood pressure 75/40) occurred about an hour following completion of the week 22 infusion of reveglucosidase alfa. Both subjects had normal blood glucose within 30 min following oral and IV dextrose. Neither subject required hospitalization for the events, and both continued treatment without dose modification. No subjects in the study required dose modification or discontinued treatment because of hypoglycemia.

Eleven of 22 subjects (50%) experienced at least one hypersensitivity or anaphylaxis AE. The most common hypersensitivity AEs were flushing (*n* = 5), urticaria (*n* = 5), erythema (*n* = 4), and pruritic rash (*n* = 3). Chest discomfort (*n* = 5), flushing (*n* = 5), and hypotension (*n* = 3) were the most common anaphylactic reactions. Seven of these subjects had AEs consistent with the National Institute of Allergy and Infectious Disease and the Food Allergy and Anaphylaxis Network criteria of anaphylaxis. Of these 7 subjects, 2 had detectable anti-drug antibodies at one or more time points through 72 weeks. Four subjects discontinued treatment because of hypersensitivity or anaphylaxis. An additional 4 subjects required temporary treatment interruption. Anaphylaxis or hypersensitivity were managed with antihistamine or steroids and did not require hospitalization.

All treated subjects tested positive for anti-reveglucosidase alfa, anti-rhGAA, anti-IGF1, and anti-IGF2 antibodies at least once during the first 72 weeks of the study. The predominant antibody responses were directed against reveglucosidase alfa and rhGAA with mean (SD) therapeutic antibody (TAb) titers of 248,710 (344981) and 262,100 (409674), respectively, at study week 72 in the 20 mg/kg cohort. Anti-reveglucosidase alfa antibodies developed more slowly in the 5 mg/kg cohort than the 10 and 20 mg/kg cohorts and the mean (SD) titer for the 20 mg/kg cohort at week 24 was 1677 (2643) (see Additional file 2). TAb titers directed against the GILT portion of reveglucosidase alfa were of much lower titer, with a mean (SD) of 967 (2718) and 1300 (3565) for IGF1 and IGF2, respectively, at study week 72. Despite the development of antibodies against IGF1 and IGF2, no overall changes in plasma levels of IGF1 or IGF2 were detected, and no sustained alterations in blood glucose levels were observed. Although anti-reveglucosidase alfa antibodies were detected in all 22 subjects, consistent associations between PK parameters and antibody titers were not detected in the higher dose cohorts. Efficacy and safety of reveglucosidase alfa were not affected by the presence of antidrug antibodies through 72 weeks.

### Efficacy

**Fig. 3:**
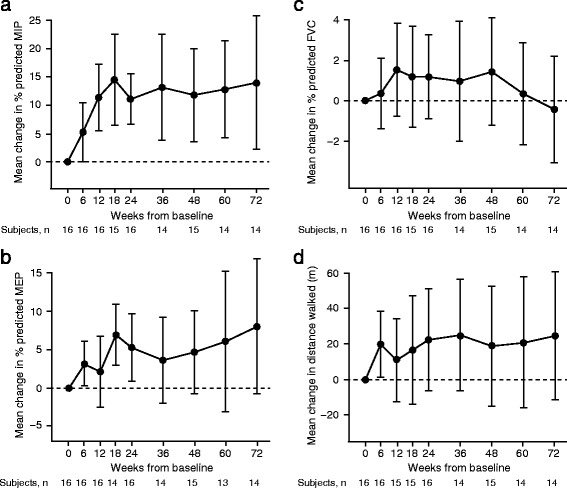
Changes in maximum inspiratory **a** and expiratory **b** pressures, forced vital capacity **c**, and distance walked **d**. Data presented as mean changes from baseline to week 72 in subjects receiving 20 mg/kg reveglucosidase alfa infusion every 2 weeks. Baseline is defined as the last measurement prior to the first infusion. The error bars represent the 95% confidence interval. The statistical significance of treatment effects was not determined, as no pre-planned statistical analyses were conducted for this study. Abbreviations: FVC, forced vital capacity; MEP, maximal expiratory pressure; MIP, maximal inspiratory pressure

**Fig. 4:**
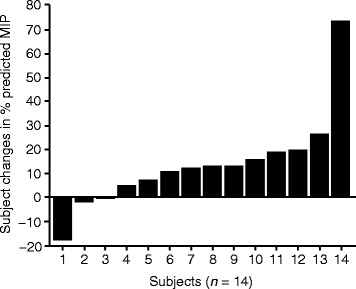
Change in % predicted MIP at week 72. Individual subject change from baseline after receiving 20 mg/kg reveglucosidase alfa intravenously every 2 weeks. Abbreviation: MIP, maximal inspiratory pressure

Positive changes from baseline were observed from 6 to 72 weeks of treatment for efficacy endpoints (MIP, MEP, FVC, 6MWT [Fig. 3] and MVV [see Additional file 3]). The greatest efficacy gains were observed early in the course of treatment with maximum improvements seen by week 18, which were maintained over the course of 72 weeks. By week 24, reveglucosidase alfa treatment increased from baseline subjects’ percent predicted MIP (mean [SD], 11.1 [8.31] %, percent predicted MEP (5.2 [8.25] %), and MVV (2.3 [10.71] L/min) values and increased the distance walked on the 6MWT (22.3 [54.23] m). This positive trend continued out to week 72 for MIP, MEP, and MVV, with mean (SD) changes from baseline of 13.9 (20.23) %, 8.0 (15.18) %, and 7.4 (25.44) L/min, respectively. At week 72, 12/14 subjects had maintained improvement in MIP (Fig. 4), indicating that MIP was increased in most subjects. In contrast, percent predicted FVC remained relatively unchanged over the course of the 72-week treatment (Fig. 3c). Results of the 6MWT for 20 mg/kg subjects were positive throughout the treatment duration (Fig. 3d), with a change of 24.5 (SD, 62.8) meters in mean absolute distance walked at week 72 compared with baseline.

## Discussion

LOPD is a rare genetic neuromuscular disorder caused by GAA deficiency, ultimately resulting in the loss of mobility and respiratory failure. Current ERT with rhGAA has limited efficacy in LOPD subjects partly because of inefficient delivery of rhGAA to skeletal muscle lysosomes and the impact of GAA deficiency in the CNS [22]. Reveglucosidase alfa is a novel rhGAA analogue with both improved lysosomal uptake [23] and glycogen reduction in skeletal muscle [19]. In this phase 1/2 study, we evaluated the PK, safety, tolerability, and efficacy of reveglucosidase alfa in ambulatory LOPD subjects who were previously untreated with rhGAA. Our results show that reveglucosidase alfa infusions were reasonably tolerated and resulted in improvement of respiratory muscle strength, and ventilatory function, however there was a limited effect on walking endurance.

The PK profile of reveglucosidase alfa is consistent with enhanced uptake of the enzyme into tissue. Reveglucosidase alfa contains a lysosomal-targeting peptide [23] that binds with high affinity to the CI-MPR, the receptor that mediates uptake of rhGAA into skeletal muscle lysosomes [9, 12, 13]. The short half-life and high CL of reveglucosidase alfa indicates a rapid distribution and suggests enhanced Cl-MPR–mediated uptake of the enzyme into tissue.

As expected, subjects developed antibodies against reveglucosidase alfa, although no impact on efficacy or safety was observed through 24 weeks. While high therapeutic antibody titers were evident at week 72, less than 20% of subjects were positive for neutralizing antibody at any time point. Moreover, despite the development of anti-IGF-IR neutralizing antibodies, overall changes in IGF-I levels were not detected in the study. Patients treated with the rhGAA alglucosidase alfa (Myozyme®; Genzyme, Cambridge, MA, USA) also develop antibodies [10, 24–27], which can block the delivery of the recombinant enzyme in tissues such as muscle and result in attenuated efficacy of Myozyme [28, 29]. Subjects treated with reveglucosidase alfa who have persistently high antibody titers would require close monitoring for efficacy and safety until the effects of the antibodies are more fully understood [13, 30].

To determine the efficacy of reveglucosidase alfa, established measures of respiratory muscle strength (MIP and MEP), lung capacity (FVC and MVV), and mobility (6MWT) were utilized [10, 27]. In particular, MIP and MEP are sensitive measures of respiratory muscle strength in patients with neuromuscular diseases [31–34] and correlate with more direct measures of diaphragmatic muscle strength. Additionally, a systematic literature review examining 174 patients with LOPD from 34 studies demonstrated that pulmonary function tests predicted ventilator use [35]. In particular, MIP and upright vital capacity consistently correlated with ventilation and wheelchair use. The current study demonstrated that reveglucosidase alfa improved MIP, MEP, MVV, and 6MWT by 24 weeks of treatment, and these improvements were maintained for >1 year of treatment, with stable lung volumes as assessed by FVC throughout the study duration. The higher gains in MIP compared with FVC may be related to changes in muscle strength occurring prior to gains in volume, in addition to potential limitations in lung volume expansion related to scoliosis and lung elasticity in neuromuscular disease [36]. The impact of scoliosis, which is common in LOPD [37], is known to contribute to extrapulmonary restrictive lung disease [38, 39]. The frequency or degree of spinal abnormalities was not evaluated in the current study.

Evaluation of the long-term benefit of reveglucosidase alfa requires accurate knowledge of the rate of disease progression in untreated patients. The natural history of LOPD suggests that respiratory muscle strength, lung capacity, and walking endurance steadily decrease at rates of 1–4% per year [1, 5, 10, 40]. van der Beek et al. [40], for example, reported annual declines in FVC (−1.1%), MIP (−3.2%), and MEP (−3.8%). The fact that reveglucosidase alfa increased respiratory muscle strength, lung capacity, and walking endurance (by as much as 13.9% for up to 72 weeks of treatment) suggests that reveglucosidase alfa has an initial effect on the disease. Longer term studies, however, are needed to determine whether reveglucosidase alfa impacts disease progression in patients with LOPD.

Given the serious clinical consequences of progressive LOPD (eg, respiratory failure and mobility loss) and other neuromuscular diseases, the benefit:risk ratio of an ERT is important to consider. In this study, a favorable benefit:risk ratio is suggested by improvements in global respiratory muscle strength and a reasonable AE profile. Although hypersensitivity-type reactions are a serious potential complication of any recombinant human protein [41], these events were manageable with antihistamines or steroids and did not require hospitalization. Hypoglycemia occurred intermittently, was mostly mild and successfully managed in all subjects using caloric supplementation shortly after each occurrence, and did not result in dose reduction or discontinuation. The reduced plasma glucose in some subjects reflects a pharmacologic effect of IGF2 derived from the GILT tag of reveglucosidase alfa [23].

## Conclusions

Additional studies are needed to further assess the safety and efficacy of this approach. Improvements in respiratory muscle strength, lung function, and walking endurance in subjects with LOPD may outbalance the risk of hypersensitivity reactions and hypoglycemia. These findings suggest that reveglucosidase alfa may provide a potential new treatment option for patients with LOPD.

## Additional files

Additional file 1: GAA activity and genotype by subject. (DOCX 13 kb)

Additional file 2: Mean anti-BMN 701 (reveglucosidase alfa) antibody titers (safety population). (DOCX 16 kb)

Additional file 3: Change from baseline in maximum voluntary ventilation (MVV). Mean changes from baseline to week 72 in subjects receiving 20 mg/kg reveglucosidase alfa infusion every 2 weeks. Baseline is defined as the last measurement prior to the first infusion. The error bars represent the standard deviations. The statistical significance of treatment effects was not determined, as no pre-planned statistical analyses were conducted for this study. (PDF 122 kb)

## Abbreviations

6MWT: 6-min walk test
AE: Adverse event
AUC: Area under the concentration-time curve
AUC_0-inf_: AUC from 0 h and extrapolated to infinity
CI-MPR: Cation-independent mannose 6-phosphate receptor
CL: Clearance
EOI: End of infusion
ERT: Enzyme replacement therapy
FVC: Forced vital capacity
GAA: Alpha glucosidase acid
GILT: Glycosylation-independent lysosomal targeting
IGF: Insulin-like growth factor
LOPD: Late-onset Pompe disease
MEP: Maximal expiratory pressure
MIP: Maximal inspiratory pressure
MVV: Maximum voluntary ventilation
PK: Pharmacokinetics
rhGAA: Recombinant human acid alpha glucosidase
SD: Standard deviation
t_½_: Terminal half-life
TAb: Therapeutic antibody
