# Supplementary material for: A study on the safety and efficacy of reveglucosidase alfa in patients with late-onset Pompe disease
Source: Orphanet J Rare Dis. 2017 Aug 24;12:144. doi: 10.1186/s13023-017-0693-2 (PMC5571484; doi:10.1186/s13023-017-0693-2)
Supplement: Supplementary file 1 — GAA activity and genotype by subject. (DOCX 13 kb) [file 13023_2017_693_MOESM1_ESM.docx]

**Additional file 1.docx** GAA activity and genotype by subject

| **Subject** | **Treatment (mg/kg)** | **GAA activity (Unit)** | **% Lower limit of normal** | **Genotype** |
| --- | --- | --- | --- | --- |
| **1** | 5 | 2 pmol/punch/hr | 6 | 1 (-45)T>G,1402 A>T |
| **2** | 5 | 6 pmol/punch/hr | 25 | c.-32-13T>G; c.2213G>A |
| **3** | 5 | 3.1 pmol/punch/hr | 31 | c.-32-13T>G and c.525delT |
| **4** | 10 | 0.1 mmol | 33 | -32-13T>G,1441T>C |
| **5** | 10 | 0.6 pmol/punch/hr | 6 | c.-32-13T>G; c.193G>T |
| **6** | 10 | 1 pmol/punch/hr | 10 | IVS1-131t>g and c.2242_2243insG |
| **7** | 20 | 14 pmol/spot/hr | 58.3 | IVS1-13t>g and c.525delT |
| **8** | 20 | 8 pmol/spot/hr | 33 | IVS1-13T>G, 2243insG |
| **9** | 20 | 0.6 nmol/hr/mg | 5.5 | c.-32.13T>G |
| **10** | 20 | 0.2 mmol/g/hr | 33 | Compound heterozygosity c,-32-13T>G and c.1548G>A p.Trp516X |
| **11** | 20 | 1.4 pmol/spot/hr | 33.2 | c.1802c>A S601X |
| **12** | 20 | 4 nmol/hr/mg | 15 | c.-32-13T>G/c 546+1G>T |
| **13** | 20 | 0.5 mU/U hexosaminid | 17.5 | Exon 18 deletion heterozygote (c.2481+102_c .2646+31del53 5)+c.-32-13t> G |
| **14** | 20 | 0.1 umol/h/L | 33 | c.-32-13T>G, c.1441T>C |
| **15** | 20 | 8.3 pmol/spot/hr | 35 | c-32-13T>G,ex on 18 deletion |
| **16** | 20 | 0.1 umol/h/L | 33 | c.-32-13T>G,c.722_723delT T |
| **17** | 20 | 0.38 umol/g/h | 12.7 | c.-32-13T>Ghterozygote c2242dupheterozygote |
| **18** | 20 | 2.1 pmol/spot/hr | 35 | intron1:c.-45T>G exon4:p.L248R |
| **19** | 20 | 8 pmol/spot/hr | 33 | IVS1-13t>g,c.525delT |
| **20** | 20 | 9 pmol/spot/hr | 18 | c.-32-13T>G heterozygote, c.2459_2461del heterozygote |
| **21** | 20 | 0.8 U/g | 8.9 | IVSO-457>G/IV S15+2T>A |
| **22** | 20 | 7 mU/U hexosaminidas | 25 | Compound heterozygosity for the pathogenic mutations c.-32-13T>G and p.2608C>T p.Arg870X |

Abbreviations: GAA, acid alpha glucosidase; hr, hour; L, liter; g, gram; U, units.
