# Supplementary material for: A study on the safety and efficacy of reveglucosidase alfa in patients with late-onset Pompe disease
Source: Orphanet J Rare Dis. 2017 Aug 24;12:144. doi: 10.1186/s13023-017-0693-2 (PMC5571484; doi:10.1186/s13023-017-0693-2)
Supplement: Supplementary file 2 — Mean anti-BMN 701 (reveglucosidase alfa) antibody titers (safety population). (DOCX 16 kb) [file 13023_2017_693_MOESM2_ESM.docx]

**Additional file 2.docx** Mean anti-BMN 701 (reveglucosidase alfa) antibody titers (safety population).


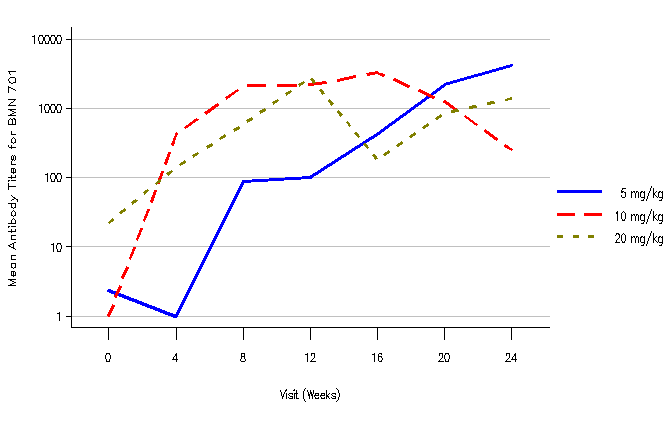


Mean antibody titers are presented by dose and time point on a semi-log_10_ scale.
