# Supplementary figures and images for: A study on the safety and efficacy of reveglucosidase alfa in patients with late-onset Pompe disease
Source: Orphanet J Rare Dis. 2017 Aug 24;12:144. doi: 10.1186/s13023-017-0693-2 (PMC5571484; doi:10.1186/s13023-017-0693-2)

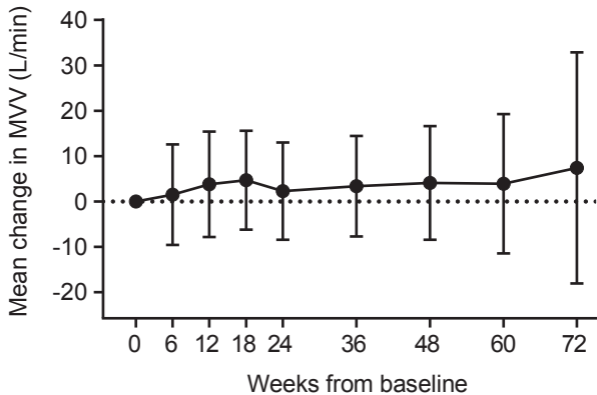

Subjects, n    16   16   16   15   15     14   15   14   14

Supplement: Supplementary file 3 — Change from baseline in maximum voluntary ventilation (MVV). Mean changes from baseline to week 72 in subjects receiving 20 mg/kg reveglucosidase alfa infusion every 2 weeks. Baseline is defined as the last measurement prior to the first infusion. The error bars represent the standard deviations. The statistical significance of treatment effects was not determined, as no pre-planned statistical analyses were conducted for this study. (PDF 122 kb) [file 13023_2017_693_MOESM3_ESM.pdf]
